# Supplementary material for: Self-Reported Cognitive Aging and Well-Being among Older Middle Eastern/Arab American Immigrants during the COVID-19 Pandemic
Source: Int J Environ Res Public Health. 2023 May 23;20(11):5918. doi: 10.3390/ijerph20115918 (PMC10252503; doi:10.3390/ijerph20115918)
Supplement: Supplementary file 1 [file ijerph-20-05918-s001.zip › ijerph-2358902-supplementary.pdf]

Table S1. Demographics Questionnaire

1. What is your date of birth: _______/_______
   Month Year

2. Are you (circle one) Male Female

3. Are you (circle one)

Single Married Divorced Separated Widowed

4. How many children do you have? ___________________

   If you have children, what are their ages?______________________________

5. How many hours of care does a family member provide, on average, per day?

   _________ hours/day

6. Do you currently live a family member that helps with your care? (circle one)

   Yes No

7. How many people, including you, live in your household currently?______________

8. In what country were you born?_______________________

   If not in U.S., what year did you arrive to U.S. ____________________

9. What is the highest grade in school you completed: _______________________

10. How would you rate your health at the present time (circle one)?

EXCELLENT FAIRLY GOOD AVERAGE NOT VERY GOOD POOR

Table S2. Demographic characteristics for each focus group.

	Focus Group 1	Focus Group 2	Focus Group 3	Focus Group 4	Focus Group 5	Focus Group 6
# of Participants	9	10*	6	8	9	3
Sex						
Male	2	7	3	0	0	0
Female	7	3	3	8	9	3
Average Range	63-86	60-88	69-90	64-69	75-88	62-77
Marital Status						
Single	1	1	0	0	1	0
Married	5	8	2	2	0	0
Divorced	0	0	0	2	1	0
Separated	0	1	0	0	0	0
Widowed	2	0	4	4	7	3
# Children	0-4	0-6	2-5	1-4	0-5	2-6
0	1	1	0	0	1	0
1	0	0	0	1	0	0
2	2	2	1	3	0	1
3	1	3	2	3	2	1
4+	4	2	3	1	6	1
Education						
Less than High school	7	5	2	2	1	2
High school	1	1	3	3	5	
College	0	4	1	3	3	1
Hours of care provided by family	0-24	0	0-2.5	0-3	0-2	0-6
Health						
Excellent	0	1	0	2	0	0
Fairly Good	0	5	2	3	5	0
Average	5	2	3	3	4	3
Not Very Good	2	0	1	0	0	0
Poor	0	0	0	0	0	0

*1 member of group signed for the gift cards but did not provide the demographics sheet so that data is missing.

Table S3. Place of birth and age of immigration.

	**Frequency**	**Percentage (%)**
**Place of Birth**		
Iraq	13	28.9
Jordan	3	6.7
Lebanon	22	48.9
Syria	1	2.2
Africa (from Lebanon)	2	4.4
Palestine	4	8.9
**Age of Immigration**		
0-18 yrs.	3	6.6
19-49 yrs.	23	51.1
50+ yrs.	19	42.2
